# Propensity score analysis the clinical characteristics of active distal and extensive ulcerative colitis: a retrospective study

**DOI:** 10.3389/fphys.2023.1136659

**Published:** 2023-06-30

**Authors:** Changchang Ge, Zhaofeng Shen, Yi Lu, Xiaojuan Liu, Yiheng Tong, Mengyuan Zhang, Yijing Liu, Hong Shen, Lei Zhu

**Affiliations:** Jiangsu Provincial Hospital of Chinese Medicine, Affiliated Hospital of Nanjing University of Chinese Medicine, Nanjing, China

**Keywords:** ulcerative colitis, propensity score, clinical characteristics, disease extent, biomarkers

## Abstract

**Background and Objectives:** Ulcerative Colitis (UC) subtypes defined by disease extent and shared pathophysiology are important. Analyzing the clinical characteristics of UC with different disease extent and optimizing clinical typing are conducive to the pathogenesis research, disease monitoring and precise treatment.

**Methods:** 188 patients with active UC were divided into distal and extensive colitis. The clinical characteristics of the two groups were analyzed by propensity score. Spearman is used for correlation analysis, and receiver operating characteristic (ROC) curve was used to evaluate the ability of clinical indicators to predict Mayo endoscopic subscore (MES).

**Results:** Compared with distal colitis, extensive colitis had more severe disease activity, younger age, higher utilization rate of corticosteroids and incidence of extra intestinal manifestations (EIMs), and clinical indicators were differentially expressed in the two groups. After using propensity score, the incidence of EIMs in the extensive colitis was still higher than that in distal colitis. Inflammation, coagulation and immune indicators like CRP, FC, IL-10, D-D and α1-MG are higher in extensive colitis, and metabolic indicators like LDL-C, HDL-C, TC, GSP and albumin are higher in distal colitis. The correlation between clinical indicators and MES is affected by disease extent. The area under curve (AUC) of CRP + D-D + α2-MG for predicting distal colitis MES3 was 0.85, and the AUC of IL-6+ GSP+ α1-MG predicted extensive colitis MES3 can reach 0.82.

**Conclusion:** Differential clinical indicators can become potential markers for predicting disease progression and prognosis, and have significance for UC mechanism research and drug development. We can select biomarkers according to lesion site.

## 1 Introduction

Ulcerative colitis is a chronic intestinal inflammatory disease. Different from Crohn’s disease (CD), which is another type of inflammatory bowel disease (IBD), UC is confined to the colon, and mucosal inflammation may extend continuously from the rectum to the proximal end as the disease progresses (Lamb et al., 2019). Severe ulcerative colitis can cause serious complications such as gastrointestinal hemorrhage, perforation and toxic megacolon, and the incidence of UC related colon cancer is significantly higher than that of the general population. Studies have found that the incidence of colorectal cancer in UC patients was 1.7 times higher than that in the control group (Damas and Abreu, 2020). Regrettably, the pathogenesis of UC is still not clear, although the clinical application of 5-aminosalicylic acid (5-ASA), hormone and biological agents have achieved good clinical results (Raine et al., 2022).

A gene association analysis study in *The Lancet* proposed a new classification of IBD based on different pathogenesis, providing a new idea for the mechanism research and clinical precision treatment of IBD (Cleynen et al., 2016).This study tested the phenotype-genotype correlation of 156,154 genetic variants in IBD population and showed that there were significant differences in the genetics between UC, colonic CD and ileal CD. Atreya and Siegmund (2021) summarized from clinical behavior, epidemiology, genetics, and intestinal microbial groups, and pointed out that there are differences between colonic and ileal CD, and colonic CD overlapped with UC in disease behavior. In addition, a great number of studies have shown that different lesion sites have different therapeutic responses to biological agents. The transverse colon presented the highest mucosa healing rate, while the right colon stenosis showed the worst improvement (Wu et al., 2020). When adalimumab was used, the ulcer changes in the rectum, sigmoid colon/left colon and transverse colon were more obvious than those in the right colon and ileum (Reinisch et al., 2017). In addition, similar conclusions were obtained in the clinical trial of vedolizumab, and he patient response to anti-integrin drugs may depend on the distribution of α4β7^+^T cells in the colon (Lobatón et al., 2014; Zundler et al., 2017; Atreya and Siegmund, 2021). This means that IBD has some heterogeneity. The simple classification of CD and UC cannot fully describe the complex IBD phenotype. Subtypes defined by lesion site and shared pathophysiology are also important and will affect treatment decisions.

Compared with CD, there are fewer studies on the site phenotype of UC. The lesion site of UC is limited to the colon and the intestinal inflammation is continuous, which is different from the jumping distribution of CD in the whole digestive tract. According to Montreal classification, UC can be divided into ulcerative proctitis (E1), left-sided colitis (E2) and extensive colitis (E3). A systematic review showed that 69.5% of UC patients had distal colitis, and patients with extensive colitis accounted for 30.5%, and the 10 year colectomy rate is 19% for those with extensive colitis, 8% with left-sided colitis and 5% with proctitis (Fumery et al., 2018). The distal colitis is not a static disease state and may continue to extend over time. The 5-year incidence of progression to extensive colitis in patients with limited UC was 17.8%, and the 10-year incidence was 31% (Roda et al., 2017). Some studies show that patients with limited lesion sites have higher risk of colectomy if the disease range is extended (Gallo et al., 2018). Although it is generally believed that patients with extensive colitis experience more severe disease activity than localized colitis, studies found no significant difference in terms of quality of life, disability index and overall cost.

Currently, some studies have focused on the potential value of clinical site phenotype in the selection of treatment regimen and pathogenesis of UC, and actively explored the predictive markers related to disease site progression (Qiu et al., 2019; Argmann et al., 2021). However, no study summarized and analyzed the clinical characteristics of distal colitis and extensive colitis. This study retrospectively analyzed the expression of clinical indicators in 188 patients with active UC from inflammation, coagulation, immunity and metabolism, and used propensity score matching the difference factors between two groups, in order to obtain the clinical characteristics of patients with distal or extensive UC, and further guide the individualized treatment and efficacy evaluation of UC, effectively slow the progress of UC.

## 2 Materials and methods

### 2.1 Study population

In this retrospective study, we collected 188 UC patients who visited the Department of Gastroenterology, Jiangsu Provincial Hospital of traditional Chinese medicine from 1 January 2019, to 30 April 2022.

This study was approved by the ethics committee of Jiangsu Provincial Hospital of Chinese Medicine from January.

### 2.2 Clinical data

All patients were diagnosed according to the combination of UC clinical, endoscopic, and histopathological criteria. Montreal classification was used to assess the lesion site, modified Truelove and Witt were used to classify the disease severity.

**Inclusion criteria**: 1) meet the diagnostic criteria of ulcerative colitis; 2) In the activity period of ulcerative colitis. **Exclusion Criteria:** 1) diseases such as shigellosis, intestinal tuberculosis, amoebiasis, Ischemic colitis and Crohn’s disease were excluded; 2) complicated with immune system diseases such as Rheumatoid Arthritis, Sjögren’s syndrome and systemic lupus erythematosus; 3) complicated with heart, brain, kidney, hematopoietic system and other important organs damage or serious infection.

Clinical information on patients with UC, including age, sex, symptoms, and colonoscopy and histopathology results, was collected from the electronic medical record system.

### 2.3 Statistical analysis

R (version 4.2.1) was used for data analysis, and the normality test was carried out for the measurement data. The measurement data conforming to normal distribution or approximate normal distribution is expressed as ‾x ± s, and two independent sample T-tests were used for analysis; nonparametric rank sum test was used for data not conforming to normal distribution. The counting data are expressed as the number of cases or the rate (%), the χ^2^ test is used for comparison. Spearman was used for multivariate correlation analysis. The difference is statistically significant if *p* < 0.05, and the difference is statistically significant if *p* < 0.01.

Propensity score was used to minimize bias and adjust for confounding factors. We used a propensity score to match the gender, age, disease duration and disease activity of the two groups. Using a greedy nearest neighbor matching approach, in which each patient in the distal colon group was matched to an extensive colon group, ultimately producing the smallest within-pair difference among all available pairs with treated patients. Patients were matched only if the Logit difference in paired propensity scores between the two groups was less than or equal to 0.5 times the pooled estimate of standard deviation.

## 3 Results

### 3.1 Clinical characteristics of included participants

A total of 188 patients with active UC were included in this study. There were significant differences in age, disease activity, incidence of EIMs and utilization rate of corticosteroids between the two groups. Patients with distal colitis were mainly mild to moderate, and patients with extensive colitis were mostly moderate to severe. Partial Mayo scores were also statistically different between the two groups, but there was no difference in MES. (Table 1).

**TABLE 1:** Characteristics of the study population and levels of Mayo score.

	Overall (n = 188)	Distal Colitis (n = 84)	Extensive Colitis (n = 104)	*p*-value
Sex, n (%)				
Male	98 (52.1%)	38 (45.2%)	60 (57.7%)	0.089
Female	90 (47.9%)	46 (54.8%)	44 (42.3%)	
Age, y (mean ± SD)	46.03 ± 14.29	49.30 ± 12.95	43.39 ± 14.82	<0.05
Disease duration (median; IQR)	4.00 (1.69–8.00)	4.00 (2.00–9.75)	3.00 (1.27 ± 7.00)	0.172
Disease activity, n (%)				
Mild	85 (45.2%)	47 (56.0%)	38 (36.5%)	<0.05
Moderate	66 (35.1%)	28 (33.3%)	38 (36.5%)	
Severe	37 (19.7%)	9 (10.7%)	28 (26.9%)	
Medications				
5-ASA	169 (89.9%)	73 (86.9%)	96 (92.3%)	0.222
Corticosteroid	31 (16.5%)	7 (8.3%)	24 (23.1%)	<0.05
Immunomodulator	3 (1.6%)	0 (0.0%)	3 (2.9%)	0.117
Biological agents	13 (6.9%)	3 (3.6%)	10 (9.6%)	0.104
Infliximab	8 (4.3%)	2 (2.4%)	6 (5.8%)	0.412
Vedolizumab	4 (2.1%)	1 (1.2%)	3 (2.9%)	
Adalimumab	1 (0.5%)	0 (0.0%)	1 (1.0%)	
Extraintestinal manifestations, n (%)	19 (10.1%)	3 (3.6%)	16 (15.4%)	<0.05
Appendectomy, n (%)	6 (3.2%)	4 (4.8%)	2 (1.9%)	0.271
Smokers, n, (%)	27 (14.4%)	12 (14.3%)	15 (14.4%)	0.979
BMI, kg/m^2^, mean ± SD	22.22 ± 3.58	22.26 ± 3.28	22.20 ± 3.80	0.09
partial Mayo score (mean ± SD), 0–9	4.30 ± 2.47	3.76 ± 2.19	4.74 ± 2.60	<0.05
MES				
1	16 (9.0%)	11 (14.3%)	5 (5.0%)	0.083
2	35 (19.8%)	16 (20.8%)	19 (19.0%)	
3	136 (71.2%)	50 (64.9%)	76 (76.0%)	

n, number; y, year; SD, standard deviation; IQR, interquartile range; BMI, body mass index; 5-ASA, 5-aminosalicylic acid; MES, Mayo endoscopic subscore; *p* values were calculated for the differences between the patients of distal colitis and extensive colitis.

### 3.2 Clinical indicators of patients with distal and extensive ulcerative colitis

#### 3.2.1 Expression of serum inflammatory markers in distal and extensive ulcerative colitis

Table 2 summarizes the expression levels of C-reactive protein (CRP), erythrocyte sedimentation rate (ESR), fecal calprotectin (FC) and serum cytokines in patients with distal and extensive ulcerative colitis. The expression of CRP, ESR, FC, interleukin-6(IL-6), IL-8 and IL-10 were different between the two groups, and except for IL-4 and IL-12P70, the expression of inflammatory markers was higher in extensive colitis.

**TABLE 2:** Expression of CRP, ESR, FC and cytokines in distal and extensive colitis.

Variable	Distal colitis	Extensive colitis	*p*-value
CRP (mg/L)	2.75 (1.65–8.82)	8.29 (3.25–25.90)	<0.01
ESR (mm/h)	14.00 (7.75–23.00)	22.00 (10.00–43.00)	<0.01
FC (ug/g)	323.10 (125.10–928.80)	875.60 (513.15–1226.15)	<0.01
IL-5 (pg/mL)	2.49 (1.56–4.36)	2.50 (1.51–3.70)	0.738
IFN-α	1.66 (1.19–2.90)	1.70 (1.04–2.43)	0.678
IL-2	1.48 (1.21–1.84)	1.58 (1.06–2.12)	0.589
IL-6	4.05 (2.34–7.91)	7.74 (3.30–13.89)	<0.01
IL-1β	3.60 (1.19–11.64)	4.34 (1.08–12.26)	0.873
IL-10	1.23 (1.00–1.67)	1.50 (1.15–2.03)	<0.05
IFN-γ	5.73 (2.79–9.46)	5.81 (2.49–12.21)	0.842
IL-8	3.26 (1.50–2.83)	6.79 (1.25–17.23)	<0.05
IL-17	2.09 (1.51–2.83)	2.13 (1.59–3.84)	0.430
IL-4	1.42 (1.12–1.69)	1.22 (0.97–1.77)	0.423
IL-12P70	1.43 (1.14–1.75)	1.37 (1.03–1.92)	0.823
TNF-α	2.46 (1.39–6.66)	2.64 (1.25–5.85)	0.729

IFN, interferon; TNF, tumor necrosis factor.

#### 3.2.2 Expression of coagulation indicators in distal and extensive ulcerative colitis

The coagulation function of UC patients is closely related to the degree of disease activity and the state of inflammation. The results in Table 3 show that the expression of D-Dimer (D-D), international normalized ratio (INR), platelet (Plt), prothrombin time (PT), fibrin degradation product (FDP) and fibrinogen (FIB) in patients with extensive colitis are significantly higher than that in distal colitis.

**TABLE 3:** Expression of coagulation related indexes in distal colitis and extensive colitis.

Variable	Distal colitis	Extensive colitis	*p*-value
D-D (mg/L)	0.36 (0.24–0.67)	0.51 (0.33–1.13)	<0.05
INR	0.98 ± 0.06	1.01 ± 0.08	<0.05
APTT (s)	39.36 ± 4.53	40.29 ± 5.39	0.209
TT (s)	17.44 ± 1.18	17.25 ± 1.33	0.307
PT (s)	13.04 ± 0.60	13.31 ± 0.79	<0.01
PT%	110.99 ± 13.71	105.93 ± 15.31	<0.05
FDP (ug/mL)	1.96 (1.40–3.02)	2.27 (1.84–3.32)	<0.01
FIB(g/L)	3.26 ± 0.81	3.88 ± 1.16	<0.01
AT (%)	94.10 ± 9.67	92.76 ± 8.88	0.332
Plt (10^9/L)	216.92 ± 93.95	267.96 ± 101.63	<0.05

APTT, activated partial thromboplastin time; TT, thrombin time; AT, antithrombin.

#### 3.2.3 Expression of serum immune indicators in distal and extensive ulcerative colitis

We next carried out a comparative analysis of the patient’s immunological indicators, mainly including complement(C), immunoglobulin (Ig) and immunoprotein electrophoresis. The results showed no significant difference in immunoglobulins between the two groups, and the serum level of C3 in distal ulcerative colitis was lower than that in extensive colitis, but there was no significant difference in C4. The results of immunoprotein electrophoresis showed that the ratio of α1- Micro globulin (MG), α2-MG, β1-MG in patients with distal colitis was also significantly lower than that in patients with extensive colitis (Table 4).

**TABLE 4:** Expression of immune indicators in distal colitis and extensive colitis.

Variable	Distal colitis	Extensive colitis	*p*-value
C3 (g/L)	0.84 ± 0.15	0.93 ± 0.18	<0.01
C4	0.23 ± 0.08	0.24 ± 0.07	0.35
IgA	2.30 ± 1.00	2.37 ± 1.14	0.66
IgG	12.65 ± 2.61	13.27 ± 3.19	0.16
IgM	1.06 ± 0.67	1.01 ± 0.43	0.56
α1-MG (%)	4.11 ± 1.04	5.16 ± 1.96	<0.01
α2-MG	9.07 ± 1.79	9.78 ± 2.43	<0.05
β1-MG	5.88 ± 0.68	6.19 ± 0.65	<0.01
β2-MG	4.69 ± 1.24	4.89 ± 1.13	0.32
γ-MG	18.34 ± 3.07	19.27 ± 3.96	0.11

#### 3.2.4 Expression of biochemical indicators in distal and extensive ulcerative colitis

We classified and compared some metabolic related indicators in the biochemical indicators, including lipid metabolism, glucose metabolism and protein metabolism (Table 5). The comparison results showed that the levels of low-density lipoprotein cholesterol (LDL-C), high-density lipoprotein cholesterol (HDL-C), apolipoprotein A1 (ApoA1), Apo E and total cholesterol (TC) in distal colitis were significantly higher than extensive colitis. In the glucose metabolism index, there was no significant difference in fasting blood glucose and HbA1c levels between the two groups, but the Glycosylated serum protein (GSP) level of distal colitis was significantly higher than that of extensive colitis.

**TABLE 5:** Expression of biochemical indicators in distal colitis and extensive colitis.

Variable	Distal colitis	Extensive colitis	*p*-value
LDL-C (mmol/L)	2.55 ± 0.68	2.25 ± 0.64	<0.01
HDL-C (mmol/L)	1.37 ± 0.37	1.15 ± 0.25	<0.01
Apo A1 (g/L)	1.33 ± 0.28	1.13 ± 0.23	<0.01
Apo E (mg/dL)	3.75 ± 1.05	3.44 ± 0.88	<0.05
TC (mmol/L)	4.34 ± 0.95	3.74 ± 0.90	<0.01
GSP (umol/L)	186.11 ± 27.93	161.88 ± 30.64	<0.01
A/G	1.69 ± 0.30	1.55 ± 0.34	<0.01
A (g/L)	41.59 ± 3.73	39.24 ± 4.79	<0.01
Pre-A (mg/L)	170.56 ± 45.54	149.94 ± 57.75	<0.01
TP (g/L)	57.90 ± 4.77	54.53 ± 6.96	<0.01

A, albumin; G, globulin; Pre-A, prealbumin; TP, total protein.

### 3.3 Comparative study of distal colitis and extensive colitis based on propensity score

From the above comparison results, it can be seen that the disease activity level of extensive colitis is higher than distal ulcerative colitis. In order to further clarify whether there are differences in clinical indicators between distal and extensive colitis, we use propensity score to balance the degree of disease activity of the two groups of patients (Table 6).

**TABLE 6:** Characteristics of the study population and levels of Mayo score after propensity score.

	Overall (*n* = 150)	Distal Colitis (*n* = 75)	Extensive Colitis (*n* = 75)	*p*-value
Sex, n (%)				
Male	82 (54.7%)	36 (48.0%)	46 (61.3%)	0.14
Female	68 (45.3%)	39 (52.0%)	29 (38.7%)	
Age, y (mean ± SD)	46.23 ± 14.50	48.55 ± 13.20	43.92 ± 15.44	0.05
Disease duration (median; IQR)	6.16 ± 6.31	6.78 ± 6.77	5.54 ± 5.80	0.229
Disease activity, n (%)				
Mild	76 (50.7%)	38 (50.7%)	38 (50.7%)	1
Moderate	56 (37.3%)	28 (37.3%)	28 (37.3%)	
Severe	18 (12.0%)	9 (12.0%)	9 (12.0%)	
Medications				
5-ASA	136 (90.7%)	66 (88.0%)	70 (51.5%)	0.262
Corticosteroid	21 (14.0%)	7 (9.3%)	14 (18.7%)	0.100
Immunomodulator	3 (2.0%)	0 (0.0%)	3 (4.0%)	0.08
Biological agents	7 (4.7%)	2 (2.7%)	5 (6.7%)	0.246
Infliximab	5 (3.3%)	2 (2.7%)	3 (4.0%)	0.520
Vedolizumab	1 (0.7%)	0 (0.0%)	1 (1.3%)	
Adalimumab	1 (0.7%)	0 (0.0%)	1 (1.3%)	
Extraintestinal manifestations, n (%)	15 (10.0%)	3 (4.0%)	12 (16.0%)	<0.05
Appendectomy, n (%)	6 (4.0%)	4 (5.3%)	2 (2.7%)	0.405
Smokers, n, (%)	23 (15.3%)	11 (14.7%)	12 (16.0%)	0.821
BMI, kg/m^2^, mean ± SD	22.25 ± 3.57	22.30 ± 3.21	22.21 ± 3.88	0.888
partial Mayo score (mean ± SD), 0–9	3.96 ± 2.32	3.95 ± 2.25	3.97 ± 2.41	0.994
MES				
1	15 (10.6%)	10 (14.5%)	5 (6.9%)	0.278
2	29 (20.6%)	12 (17.4%)	17 (23.6%)	
3	97 (68.8%)	47 (68.1%)	50 (69.4%)	

n, number; y, year; SD, standard deviation; IQR, interquartile range; BMI, body mass index; 5-ASA, 5-aminosalicylic acid; MES, Mayo endoscopic subscore; *p* values were calculated for the differences between the patients of distal colitis and extensive colitis.

The results showed that after excluding the influence of disease activity factors, there was no difference in age, utilization rate of corticosteroids and pMayo between the two groups, but the incidence of EIMs in extensive colitis was still higher than that in distal colitis. Although some clinical objective indicators have become similar after balanced treatment with propensity score (ESR, IL-6, IL-8, Pt, FDP, etc.), there are still many other indicators that are differentially expressed between the two groups (Table 7).

**TABLE 7:** Difference of clinical indexes between distal and extensive ulcerative colitis after propensity score.

Variable	Distal colitis	Extensive colitis	*p*-value
CRP (mg/L)	2.77 (1.47–9.11)	6.00 (2.90–12.33)	0.004
FC (ug/g)	459.30 (125.07–967.78)	865.20 (307.55–1263.90)	0.012
IL-10 (pg/mL)	1.22 (1.00–1.69)	1.49 (1.15–2.02)	0.045
D-D (mg/L)	0.36 (0.24–0.69)	0.48 (0.31–0.95)	0.045
FIB(g/L)	3.32 ± 0.84	3.70 ± 1.09	0.021
C3 (g/L)	0.84 ± 0.16	0.92 ± 0.17	0.009
A/G	1.69 ± 0.31	1.58 ± 0.32	0.049
A (g/L)	41.50 ± 3.79	40.03 ± 4.19	0.026
LDL-C (mmol/L)	2.54 ± 0.68	2.23 ± 0.62	0.006
HDL-C (mmol/L)	1.33 ± 0.35	1.19 ± 0.26	0.013
TC (mmol/L)	4.30 ± 0.95	3.77 ± 0.91	0.001
GSP (umol/L)	184.54 ± 28.72	168.23 ± 29.57	0.002
Apo A1 (g/L)	1.30 ± 0.27	1.17 ± 0.23	0.003
α1-MG (%)	4.21 ± 1.06	4.70 ± 1.54	0.046
β1-MG (%)	5.90 ± 0.67	6.24 ± 0.67	0.007

### 3.4 The value of clinical indicators in distal and extensive ulcerative colitis is different

#### 3.4.1 Correlation between clinical indexes and MES in distal and extensive ulcerative colitis

**FIGURE 1:**
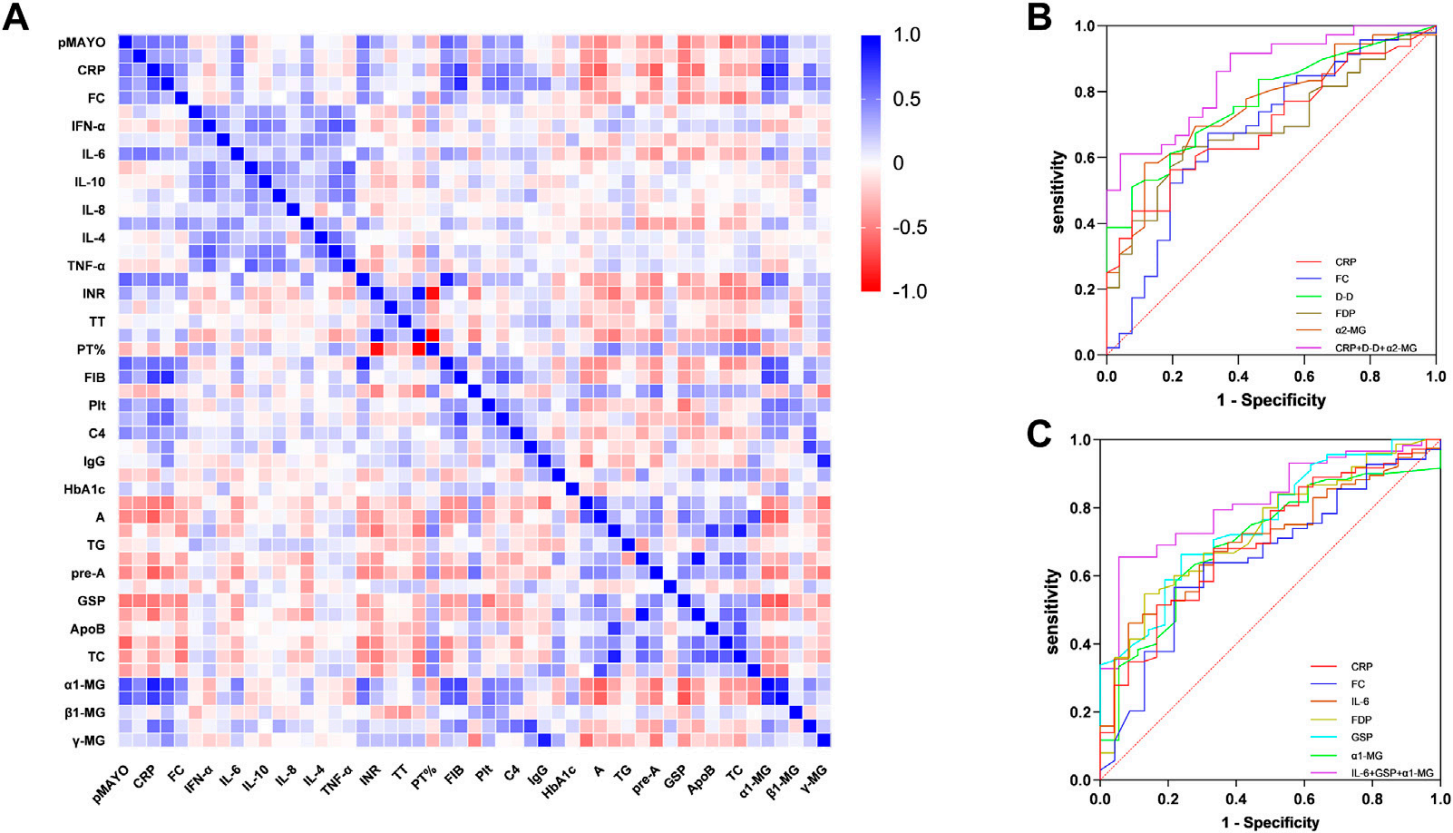
**(A)** Spearman correlation analysis between Mayo score and clinical objective indexes; **(B)** ROC curve of clinical biomarkers predicting MES 3 in active distal ulcerative colitis; **(C)** Figure 3 ROC curve of clinical biomarkers predicting MES 3 in active extensive ulcerative colitis.

Patients with distal and extensive ulcerative colitis have different disease activity, and this difference significantly affects the expression of clinical markers. We performed Spearman correlation analysis for the above clinical indicators. (Figure 1A). There is no doubt that MES and pMayo, as the scores reflecting the activity of UC, have a good correlation with CRP, ESR, FC and IL-6. The correlation between α1-MG and pMayo was the highest (r = 0.698), and the correlation between α1-MG and CRP was as high as 0.867. This suggests that α1-MG may serve as a potential marker for predicting ulcerative colitis disease activity.

We selected the indicators with strong correlation with MES for group analysis. The results showed that there was no correlation between ESR and MES in both groups of patients, while IL-6 and GSP were only correlated with MES in extensive colitis and not in distal colitis. CRP, FC, D-D, FDP, a1-MG and a2-MG were correlated with MES in the two groups. Although the correlation coefficients were different, there was no statistical difference between the two groups (Table 8).

**TABLE 8:** Correlation analysis of clinical indexes and MES between the two groups.

	Distal colitis		Extensive colitis		*p*-value
	r	*p*-value	R	*p*-value	
CRP	0.349**	0.002	0.308**	0.002	0.76
ESR	0.134	0.264	0.110	0.289	—
FC	0.339**	0.004	0.214*	0.041	0.38
IL-6	0.220	0.054	0.304**	0.002	—
D-D	0.458**	0.000	0.221*	0.029	0.07
FDP	0.327**	0.004	0.346**	0.000	0.89
GSP	−0.217	0.071	−0.379**	0.000	—
a1-MG	0.484**	0.000	0.304**	0.007	0.20
a2-MG	0.383**	0.002	0.244*	0.032	0.36

**p<0.01 and *p<0.05.

#### 3.4.2 The ability of clinical indicators to predict MES 3 in patients with distal and extensive colitis

We further analyzed the ability of clinical indicators to predict MES 3 in both groups of patients, and the research results are presented as ROC curves in Figures 1A, B. In patients with distal ulcerative colitis, the AUC of CRP, FC, D-D, FDP, and α2-MG independently predicted MES 3 is 0.70, 0.68, 0.77, 0.69, and 0.76, respectively, and the AUC of CRP + D-D + α2-MG for predicting distal colitis MES 3 was 0.85, with a sensitivity and specificity of 61% and 96%, respectively.

In patients with extensive colitis, the AUC of CRP, FC, IL-6, FDP, GSP and α1-MG independently predicted MES 3 is 0.70, 0.65, 0.70, 0.73, 0.76, and 0.70, respectively, and the AUC of IL-6 + GSP+ α1-MG predicted MES3 can reach 0.82, with sensitivity and specificity of 66% and 94%, respectively (Figures 1B, C).

## 4 Discussion

The site phenotype of ulcerative colitis is dynamic and closely related to the disease activity, which suggests that we cannot judge the disease condition based on the initial lesion site, so dynamic follow-up is required during treatment. In this study, we divided the patients into two groups: distal and extensive ulcerative colitis, and compared their clinical characteristics and objective indicators.

The results showed that the age, disease activity and the incidence of EIMs were in great differences between the two groups. These three are interrelated, and were listed as high-risk characteristics of ulcerative colitis in the 2019 ACG and AGA adult UC guidelines, emphasizing more active treatment in the early stage (Ko et al., 2019; Rubin et al., 2019). In the second part, we homogenized the disease activity of the two groups and found that when the disease activity was the same, the incidence of EIMs in extensive colitis was still higher than those with distal colitis. Therefore, EIMS are often considered to be inflammation located outside the gut of IBD patients, and their pathogenesis depends on the extension of intestinal immune response and has a common environmental or genetic predisposition with IBD (Hedin et al., 2019).

Biomarkers for disease activity monitoring and treatment response have been a notable topic for research of UC in recent years. In this study, we incorporated the clinical objective indicators including cytokines as much as possible. In the first part of the study, we found that a large number of clinical indicators were differentially expressed in the two groups. Most of these clinical indicators have been confirmed to be related to the disease activity of ulcerative colitis in previous studies (Kato et al., 2016; Langhorst et al., 2016; Sollelis et al., 2019; Ge et al., 2022). In order to exclude the influence of clinical markers of disease activity, the analyses were adjusted by the propensity score. CRP, ESR and FC are the most commonly used indicators to reflect the disease activity of UC, so why do CRP and FC levels have significant differences when the disease activity is the same? Compared with distal colitis, patients with extensive colitis have a wider range of intestinal lesions, and the above results suggest that CRP and FC are more sensitive than ESR in responding to intestinal inflammation. Thus, elevated levels of CRP and FC in patients with distal colitis may serve as potential markers to predict progression of the lesion. Similarly, the expression of D-D, FIB, C3, α1-MG, β1-MG also has the potential to become biomarkers.

The abnormal immune response of intestinal mucosa is an important internal factor causing inflammation and tissue damage in UC (Leppkes and Neurath, 2020). Cytokines play an important role in mediating the abnormal immune response. Compared with non -specific inflammatory indicators such as CRP, cytokines can be used as biomarkers to respond to intestinal inflammation, and also closely related to IBD mechanism research and drug research and development. As of now, the clinical treatment of IBD has entered the era of biological agents, but the research status of new biological agents are not satisfactory. Our results suggest that IL-10 expression in patients with extensive colitis is significantly higher than that in patients with distal colitis when disease activity is similar between the two groups. IL-10 is generated by CD4+TH2 sub-groups, and is also known as cytokine synthesis inhibitory factor (CSIF). It can inhibit macrophages to secrete cytokines and have extensive immune regulation activity. Studies have shown that IL-10 knockout mice have spontaneous enteritis, suggesting that the occurrence of UC is related to the reduction of IL-10. However, when inflammation occurs, the expression of IL-10 in the inflammatory and non-inflammatory areas of intestinal mucosa is significantly increased to play an anti-inflammatory role (Melgar et al., 2003). In addition, studies show IL-10 and IL-10R1 levels were increased in transverse colon biopsies of patients with extensive/pancolitis, compared with control subjects and patients with limited distal disease (Wittmann Dayagi et al., 2021). On 6 July 2022, Applied Molecular Transport (AMT) announced the results of the 2-stage MARKET test IL-10 inhibitor in patients with moderate to severe ulcerative colitis. The combination of IL-10 inhibitor and adalimumab did not show better clinical efficacy compared to that of adalimumab monotherapy. But, for patients with UC less than 5 years, the clinical remission rate of patients receiving combination therapy was 43.8%, while that of patients receiving adalimumab alone was 15.4%, suggesting that combination therapy may be beneficial in the early stage of the disease (Applied Molecular Transport, 2022). These results all indicate that IL-10 has great potential in the clinical study of UC.

TC, LDL-C, HDL-C, Albumin, GSP and other metabolic markers were also differentially expressed in the two groups. The metabolic process of the body is regulated by many physiological and pathological links. The dysregulation of intestinal flora is related to a variety of human metabolic diseases. Studies have found that the amount and types of beneficial bacteria which has important and unique significance for the integrity of intestinal mucosal barrier are significantly reduced in the gut of IBD patients. (Turnbaugh et al., 2009; Henao-Mejia et al., 2012; Leung et al., 2016; Vatanen et al., 2018). Intestinal flora can produce a great number of metabolites in the colon that can affect the physiological process of the body. Gut microbiota-derived metabolites were also recognized as key actors in IBD (Lavelle and Sokol, 2020). The results showed that the metabolic indexes of patients with extensive colitis were lower than those with distal colitis, among which lipid metabolism were the most obvious. This difference is closely related to the proximal colonic inflammation. Take lipid metabolism as an example, bile acids are the most widely studied gut microbiota-derived metabolites. Studies have shown that bile acid is the main way to decompose metabolism. In the state of inflammation, bile acid metabolism rises to promote inflammation. The intestinal flora can also play an important role in regulating dietary fat absorption and lipid metabolism by affecting bile acid metabolism, generating short -chain fatty acids, and regulating intestinal endocrine systems (Yu et al., 2019). When the expression of bile acid in the inflammation area is increased, the synthesis of cholesterol will be reduced accordingly, which can reflect intestinal inflammation. Dongke Xu’s study indicated that the Cholesterol Sulfate (CS) can promote the biological synthesis of cholesterol, thereby alleviating ulcerative colitis (Xu et al., 2022). Total cholesterol levels can be used as a short-term therapeutic target. Maiko et al. demonstrated that during induction therapy in patients with acute ulcerative colitis, patients with mucosal healing had higher white blood cell counts and total cholesterol than those without (Motobayashi et al., 2019).

A large number of studies have focused on clinical markers used to predict disease activity, but few have examined whether the predictive value of different biomarkers is influenced differently by lesion site. To this end, we preliminarily explored the correlation between clinical biomarkers and MES in two groups. ESR was associated with MES overall, but after grouping ESR was not associated with MES in either distal colitis or extensive colitis. Furthermore, IL-6 and GSP were associated with MES in extensive colitis but not in distal colitis. CRP, FC, D-D, FDP, a1-MG and a2-MG were correlated with MES in the two groups. Although the correlation coefficients were different, there was no statistical difference between the two groups. In addition, the ability of biomarkers to predict MES 3 in the two groups are different. The lesion site of ulcerative colitis may affect the predictive ability of biomarkers.

In conclusion, this study conducted a comparative analysis of the clinical characteristics and objective indicators of distal colitis and extensive colitis, with the aim of finding biomarkers related to disease site extension and exploring the significance of different indicators in predicting disease activity, mechanism research and drug development. However, this study is only a preliminary exploration, subsequent large-scale retrospective studies and prospective clinical studies are needed to further verify the results.

## Authors’ note

The manuscript, including related data, figures and tables has not been previously published and that the manuscript is not under consideration elsewhere.

## Data Availability

The raw data supporting the conclusion of this article will be made available by the authors, without undue reservation.
